# Gefitinib and Methotrexate to Treat Ectopic Pregnancies with a Pre-Treatment Serum hCG 1000–10,000 IU/L: Phase II Open Label, Single Arm Multi-Centre Trial^☆^

**DOI:** 10.1016/j.ebiom.2018.06.017

**Published:** 2018-06-22

**Authors:** Monika M. Skubisz, Stephen Tong, Ann Doust, Jill Mollison, Terrance G. Johns, Peter Neil, Miranda Robinson, Siladitya Bhattacharya, Euan Wallace, Nicole Krzys, W. Colin Duncan, Andrew W. Horne

**Affiliations:** a Translational Obstetrics Group, Department of Obstetrics and Gynaecology, University of Melbourne, Heidelberg 3084, Victoria, Australia; b Mercy Perinatal, Mercy Hospital for Women, Heidelberg 3084, Victoria, Australia; c The Hudson Institute, Clayton 3168, Australia; d Monash Health, Clayton 3168, Victoria, Australia; e MRC Centre for Reproductive Health, University of Edinburgh, Edinburgh EH16 4TJ, United Kingdom; f University of Oxford, Nuffield Department of Primary Health Sciences, Oxford OX2 6GG, United Kingdom; g Institute of Applied Health Sciences, University of Aberdeen, Aberdeen AB25 2ZD, United Kingdom

**Keywords:** Gefitinib, Methotrexate, Ectopic pregnancy, Medical treatment, Phase II

## Abstract

**Background:**

Ectopic pregnancies are a leading cause of maternal mortality. Most are treated surgically. We evaluated the efficacy and safety of combining oral gefitinib (epidermal growth factor receptor inhibitor) with methotrexate to treat larger ectopic pregnancies.

**Methods:**

We performed a phase II, single arm, open label study across four hospitals in Edinburgh and Melbourne. We recruited women with a stable tubal ectopic pregnancy and a pre-treatment serum hCG between 1000 and 10,000 IU/L. We administered intramuscular methotrexate (50 mg/m^2^) once, and oral gefitinib (250 mg) for seven days. The primary outcome was the percentage successfully treated without needing surgery. To show the treatment is at least 70% effective, 28 participants were required, and 24 or more successfully treated without surgery. Secondary outcomes were safety, tolerability, and time to resolution. This study is registered (ACTRN12611001056987).

**Findings:**

30 participants with stable tubal ectopic pregnancies were recruited but two withdrew, leaving 28 participants. The median (± range) pre-treatment serum hCG was 2039 (1031–8575) IU/L and nine had pre-treatment hCGs levels >3000 IU/L. The treatment successfully resolved 86% (24/28) cases with a median (±range) time to resolution of 32 (18–67) days. The treatment caused transient rash and diarrhoea, but no serious adverse events.

**Interpretation:**

Combination gefitinib and methotrexate is at least 70% effective in resolving ectopic pregnancies with a pre-treatment serum hCG 1000–10,000 IU/L. This may be a new way to treat most stable ectopic pregnancies, but needs to be validated via a randomised clinical trial.

## Highlights

• Methotrexate and seven tablets of gefitinib resolved 86% of ectopic pregnancies with a starting hCG 1000-10,000 IU/L.
• The treatment commonly caused a transient rash and diarrhea, but no serious adverse events
• This could be more effective than methotrexate alone to treat ectopic pregnancies, allowing more to avoid surgery.

Evidence before this study: We searched PubMed for articles before May 31st without restriction on the start date. We included the search terms “ectopic pregnancy”, AND “treatments” AND “gefitinib”. There is already an extensive literature evaluating the use of methotrexate to treat ectopic pregnancies, and reviewing each of these trials was beyond the remit of our current study. Hence for methotrexate treatment we examined meta-analyses', reviews and international clinical guidelines on the medical management of ectopic pregnancies. We also searched for general reviews on the topic of ectopic pregnancy treatment (search terms “ectopic pregnancy” AND “review”).

Most ectopic pregnancies are treated surgically. An injection of methotrexate is now widely used clinically to treat ectopic pregnancy. However, the efficacy of methotrexate declines with increasing pre-treatment serum human chorionic gonadotrophin (hCG) levels, and declines when other clinical parameters are present suggesting the ectopic pregnancy is large (demonstrable fetal cardiac activity or a large size seen on ultrasound). One systematic meta-analysis published 10 years ago concluded that methotrexate treatment is only more cost economical than surgery if the pre-treatment serum hCG is <1500 IU/L.

A preclinical laboratory study identified the possibility that combining gefitinib (an orally available epidermal growth factor receptor inhibitor) with methotrexate may be additive in treating ectopic pregnancies, compared to either agent alone. There have also been two early phase clinical studies published. The first was a trial of 12 participants where the inclusion criteria was an ectopic pregnancy with a pre-treatment hCG <3000 IU/L. It showed the treatment appeared safe, and a comparison with a historic cohort of women treated with methotrexate alone suggested adding gefitinib may induce faster declines in serum hCG levels and resolves the ectopic pregnancy faster. The second human study was a case series that showed combination gefitinib and methotrexate resolved eight cases of extra-tubal ectopic pregnancies.

Added value of this study: This is the first study evaluating the potential of combination gefitinib and methotrexate in treating tubal ectopic pregnancies presenting with pre-treatment hCG levels between 1000 and 10,000 IU/L. This single arm clinical trial of 28 participants found combining seven tablets of gefitinib with methotrexate resolved 86% of ectopic pregnancies in a cohort that included larger ectopic pregnancies.

Implications of all the available evidence: Collectively, three single arm human trials suggest combination gefitinib and methotrexate may be a new medical treatment to resolve most ectopic pregnancies. However, the concept now needs to be tested in large randomised clinical trial before it can be used clinically.

**Research in context**

**Evidence before this study**

We searched PubMed for articles before May 31st without restriction on the start date. We included the search terms “ectopic pregnancy”, AND “treatments” AND “gefitinib”. There is already an extensive literature evaluating the use of methotrexate to treat ectopic pregnancies, and reviewing each of these trials was beyond the remit of our current study. Hence for methotrexate treatment we examined meta-analyses’, reviews and international clinical guidelines on the medical management of ectopic pregnancies. We also searched for general reviews on the topic of ectopic pregnancy treatment (search terms “ectopic pregnancy” AND “review).

Most ectopic pregnancies are treated surgically. An injection of methotrexate is now widely used clinically to treat ectopic pregnancy. However, the efficacy of methotrexate declines with increasing pre-treatment serum human chorionic gonadotrophin (hCG) levels, and declines in the presence when other clinical parameters are present that suggests the presence of a large ectopic pregnancy (demonstrable fetal cardiac activity or a large size seen on ultrasound). One systematic meta-analysis published 10 years ago concluded that methotrexate treatment is only more cost economical than surgery if the pre-treatment serum hCG is <1500 IU/L.

A preclinical laboratory study identified the possibility that combining gefitinib (an orally available epidermal growth factor receptor inhibitor) with methotrexate may be additive in treating ectopic pregnancies, compared to either agent alone. There have also been two early phase clinical studies published. The first was a trial of 12 participants where the inclusion criteria was an ectopic pregnancy with a pre-treatment hCG <3000 IU/L. It showed the treatment appeared safe, and a comparison with a historic cohort of women treated with methotrexate alone suggested adding gefitinib may induce faster declines in serum hCG levels and resolves the ectopic pregnancy faster. The second human study was a case series that showed combination gefitinib and methotrexate resolved eight cases of extra-tubal ectopic pregnancies.

**Added Value of This Study**

This is the first study evaluating the potential of combination gefitinib and methotrexate in treating tubal ectopic pregnancies presenting with pre-treatment hCG levels between 1000-10,000 IU/L. This single arm clinical trial of 28 participants found combining seven tablets of gefitinib with methotrexate resolved 86% of ectopic pregnancies in a cohort that included larger ectopic pregnancies.

**Implications of all the Available Evidence**

Collectively, three single arm human trials suggest combination gefitinib and methotrexate may be a new medical treatment to resolve most ectopic pregnancies. However, the concept now needs to be tested in large randomized clinical trial before it can be used clinically.

## 1 Introduction

Ectopic pregnancy complicates 1–2% of pregnancies [1] and is the most common life-threatening condition in early pregnancy. In the United Kingdom, there are 12,000 cases of ectopic pregnancy every year and they contribute to 3–8% of all maternal pregnancy related deaths [2]. 98% are tubal ectopic pregnancies where the pregnancy implants in the Fallopian tube.

Ectopic pregnancies can be treated surgically (mainly by operative laparoscopic excision), or medically (intramuscular injection of the folate antagonist, methotrexate, followed by serial monitoring of serum hCG concentrations) [3]. However, the efficacy of methotrexate treatment is lower with higher pre-treatment serum hCG concentrations [3]. Hence, many cases are still treated surgically [3] and there is a need for a more effective medical therapy to reduce operative intervention (and its inherent risks) in women diagnosed with ectopic pregnancy.

In many fields of medicine, notably oncology and rheumatology (where methotrexate is in widespread clinical use), it is clear that outcomes are improved with combination treatments which target different aetiological pathways, compared to single agent treatment. We propose that the addition of oral gefitinib (an epidermal growth factor receptor [EGFR] antagonist) to the current medical management regimen of intramuscular methotrexate could provide an exciting clinical solution to the suboptimal medical therapy currently available for the management of ectopic pregnancy. Gefitinib is a molecularly targeted drug that blocks EGFR signalling, and is licensed to treat non-small-cell lung cancer.

In preclinical studies, we have shown that ectopic pregnancy implantation sites (trophoblast cells) express high levels of EGFR and that gefitinib augments methotrexate-induced regression of pregnancy-like tissue [4]. Importantly, the two agents work additively in trophoblast cells to potently inhibit cell growth, block EGFR signalling pathways, and enhance apoptosis.

We previously reported a Phase I (Gefitinib and Methotrexate Trial 1, or GEM1) single-arm open-label dose-escalation study administering a combination of intramuscular methotrexate (50 mg/m^2^, standard care) and 250 mg oral gefitinib (one dose (n = 3), three daily doses (n = 3), seven daily doses (n = 6)) to 12 women with ectopic pregnancy (serum hCG <3000 IU/L) [5]. The combination of methotrexate and gefitinib did not cause any significant toxicities (assessed clinically and by serial biochemical assessment) or serious side effects. We have also reported a case series where we successfully treated eight extra-tubal ectopic pregnancies with gefitinib and methotrexate [6]. While preliminary, the collective data from the preclinical work and the two small early trials suggest combination methotrexate and gefitinib merit further consideration as an effective medical treatment for ectopic pregnancy.

We therefore undertook a phase II clinical trial. Importantly, we wanted to examine whether the treatment is efficacious in treating larger tubal ectopic pregnancies where current medical management is more likely to fail. We set out to recruit tubal ectopic pregnancies with pre-treatment serum hCG concentrations between 1000 and 10,000 IU/L.

## 2 Materials and Methods

We conducted a phase II single-arm multi-centre open label trial to examine the efficacy and safety of a single dose of intramuscular methotrexate and daily oral gefitinib for seven days to treat tubal ectopic pregnancies (Trial registration number: ACTRN12611001056987). We named this the GEM (Gefitinib and Methotrexate) II study and the protocol has previously been published [7]. This was an investigator led project and the funders had no role in the conduct of the study.

In women with stable tubal ectopic pregnancy with hCG concentrations between 1000 and 10,000 IU/L we expected the success of methotrexate treatment (defined as a decline in serum hCG < 15 IU/L without the need for surgery) to be 70% or less. Using A'Hern's formula for Phase II one-stage designs [8], with 80% power and a 5% level of significance, 28 patients were required to enable us to assess whether the proportion of patients with a successful outcome to treatment was >70%. The reason we selected this figure is that methotrexate in the clinic appears to have a success rate of around 70% [9, 10]. Our power calculation found if 24 or more patients have a successful outcome, we can reject the hypothesis that the true efficacy of combination gefitinib and methotrexate is ≤70%.

We recruited women presenting with tubal ectopic pregnancies with a pre-treatment serum hCG concentration between 1000 and 10,000 IU/L who were considered clinically stable at four hospitals: Royal Infirmary of Edinburgh (Edinburgh, United Kingdom), Mercy Hospital for Women (Victoria, Australia) and two hospitals within the Monash Health Network (Monash Medical Centre and Dandenong District Hospital, both in Victoria, Australia). Ethical approval was obtained from the Scotland A Research Ethics Committee (LREC 12/SS/0005) (UK), the Southern Health Human Research Ethics Committee B (SH HREC 11180B) and the Mercy Health Human Research Ethics Committee (R12/25). We obtained written, informed consent for all participants.

Our inclusion criteria were: women aged between 18 and 45 years; pre-treatment serum hCG of 1000–10,000 IU/L (rising or static); ultrasound diagnosis of definite tubal ectopic pregnancy (extrauterine gestational sac with yolk sac and/or embryo, with or without cardiac activity) or probable (inhomogeneous adnexal mass or extra-uterine sac-like structure) performed by a clinical team of trained, qualified and experienced ultrasonographers; no clinical evidence of intra-abdominal bleeding; no pallor; no guarding/rigidity on abdominal examination; stable blood pressure and heart rate; haemoglobin on full blood examination at day 1 between 100 and 165 g/L.

Our exclusion criteria were: women with a pregnancy of unknown location; evidence of a significant intra-abdominal bleed on ultrasound defined by free fluid above the uterine fundus or surrounding the ovary; women with a history of any significant pulmonary disease; abnormal liver/renal/haematological indices; significant pre-existing dermatological conditions; significant pre-existing gastrointestinal medical illnesses; and Japanese ethnicity (as those of Japanese descent who are administered gefitinib have been reported to be at higher risk of developing interstitial lung disease) [11].

Our intervention was a single dose intramuscular methotrexate (50 mg/m^2^) injection with seven daily doses oral gefitinib (250 mg). We started the administration of gefitinib on the same day that the first methotrexate injection was given. To monitor treatment response, we followed protocols to track serial serum hCG concentrations widely used for medical management with methotrexate, and first proposed by Stovall et al. [12]. Serum hCG levels were measured on days 4, 7 and 11, then weekly until hCG levels declined to non-pregnant levels (<15 IU/L). All women were reviewed regularly and subsequent management, and decision for surgery, was based on normal clinical care. Surgery was considered if there was a persistent lack of response to the treatment (evidence of a lack of a fall in serum hCG concentrations) or there was clinical evidence raising suspicions of active bleeding or tubal rupture.

To monitor safety and tolerability, women were assessed clinically (history) and biochemically (haematological, renal and liver function tests) on days 4 and 7 (or if elevated, they were offered repeat testing until any abnormal values returned to normal physiological levels).

Our primary outcome was the resolution of the tubal ectopic pregnancy without the need for surgery. Resolution was defined by serum hCG concentrations (the current clinical marker to monitor treatment response) falling to non-pregnant levels (hCG <15 IU/L, which corresponds to a negative urinary pregnancy test using the most sensitive assays). Failure was therefore defined as women who required salvage surgery.

Our second outcome was to document safety, tolerability and adverse events, as determined by clinical and biochemical assessment. Patients were regularly reviewed clinically and we confirmed normal renal, liver and haematological function tests on day 1 and assessed treatment effects on days 4 and 7. Furthermore, participants were asked to collect information about adverse events in treatment diaries. They were instructed to contact the clinical research team at any time after consenting to join the trial if they have an event that requires hospitalisation or an event that results in persistent or significant disability or incapacity. The protocols and evaluation in place for serious adverse event reporting are described in detail in the published study protocol [9]. After treatment, participants were contacted at least 3 and 6 months post treatment to document return of menstrual cycles and any subsequent pregnancies.

We also compared the numbers who were successfully treated without need for surgery to women not enrolled in the trial, but presented to our clinical services during the period the trial was recruiting participants and treated with methotrexate alone. These were women diagnosed with tubal ectopic pregnancies presenting at our hospitals with similar pre-treatment serum hCG levels during the time period while we were recruiting for the trial, but either declined to participate in our trial (or were not approached) and were treated with methotrexate instead.

Given this was a single arm efficacy trial, the data were expressed using descriptive statistics. Comparisons of serum hCG levels were done using the Mann-Whitney *U* test. Analysis and reporting followed CONSORT guidelines. This was an investigator initiated trial, with no funding from industry. ST, MMS and AWH had full access to all the data in the study and had final responsibility for the decision to submit the data for publication.

## 3 Results

**Fig. 1:**
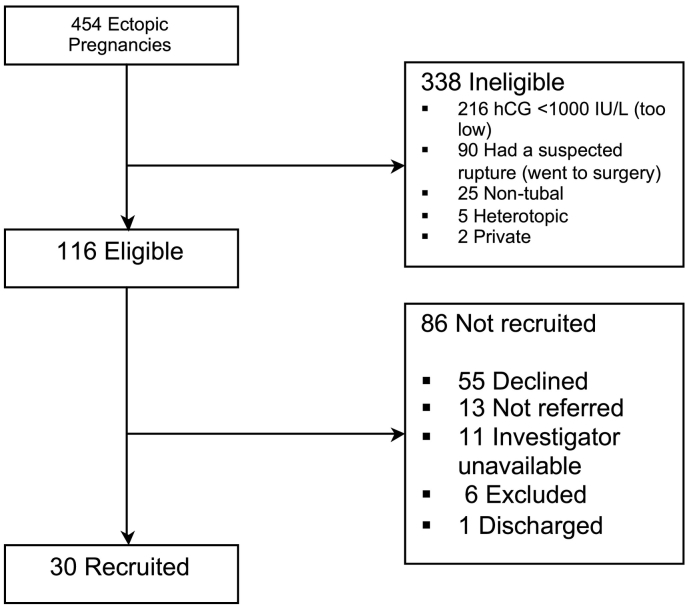
Flow of participants recruited to the study.

We recruited 30 women diagnosed with stable tubal ectopic pregnancies with a pre-treatment serum hCG 1000–10,000 IU/L presenting between January 2012 and April 2014. Fig. 1 shows the flow of participants.

Two participants withdrew soon after commencing treatment because they decided that they wanted surgical management (despite adequately falling serum hCG levels and no clinical evidence of tubal rupture i.e. not due to evidence of treatment failure). As we did not plan an intention to treat analysis, they were excluded from the analysis and we continued to recruit participants until we reached our target of 28 cases.

The clinical baseline details of the 28 participants who met inclusion criteria and participated in the study are shown in Table 1. The median (range) pre- treatment serum hCG concentrations were 2039 (1031–8675) IU/L. Nine cases (32%) had a pre-treatment serum hCG concentration above 3000 IU/L.

**Table 1:** Baseline characteristics (n = 28).

Characteristic	
Age – (median and IQR)	30.5 (25.5–33.8)
Previous live births – n (%)	17 (61%)
Body mass index – (median and IQR)	27.0 (23.0–30.3)
Previous ectopic pregnancy - n (%)	6 (31%)
Self reported past history of pelvic infection	4 (14%)
Presence of a sexually transmitted infection - n (%)^a^	1 (4%)
Conception by assisted reproductive technology – n (%)	4 (14%)
Smoker	
Never	15 (54%)
Past smoker	6 (21%)
Current smoker	7 (25%)

Body mass index was not recorded in 2 cases. IQR – Interquartile range.

a 23 out of the 28 participants were screened at recruitment for the presence of sexually transmitted infections.

The ectopic pregnancies were successfully resolved with combination therapy in 24 participants (86%), the remaining four were offered surgery. There was a significant difference in pre-treatment hCG concentrations between the four requiring surgery (median 3500 (range 2876–7551) IU/L and the 24 successfully treated (median serum hCG concentrations 1922 (range 1030–8575) IU/L; p = 0.012, Mann-Whitney-*U* test)). There was only one case recruited where there was fetal cardiac activity. The pre-treatment hCG concentration was 7551 IU/L and it was among the four cases that required surgery.

Among the 28 participants, there were 19 cases with a serum hCG 1000–3000 IU/L and of these, 18 were successfully treated (94.7% success rate). Thus, most of the cases where salvage surgery was required were those where the pre-treatment serum hCG were above 3000 IU/L.

The treatment appeared safe and there were no reported serious adverse events attributable to the medication and no biochemical abnormalities seen on serial haematological and renal function testing. A transient rash occurred in 16 (57%) of participants and 13 had diarrhoea, known side effects of gefitinib. Six participants had mild transient elevations of serum liver enzyme levels (either elevated Aspartate aminotransferase or Alanine aminotransferase levels, a possible side effect of methotrexate) that then normalised with completion of the treatment. Other non-serious side effects reported that may, or may not be attributable to the trial medication were: 15 with nausea (2 reported vomiting), 10 reported lethargy, 8 had dizziness and 7 had pruritus.

Thus, none of our three single arm trials (the current report and our prior early phase trials [5,6] identified a serious adverse event that was likely to be attributed to the medication. A transient skin rash has been noted to occur commonly (57% in this trial and 67% in our previous phase I trial [5]) as well as diarrhoea and these are likely to be common sides effects of this treatment.

We also obtained data from a contemporaneous cohort of 32 women diagnosed with a tubal ectopic pregnancy (with a pre-treatment serum hCG concentration between 1000 and 10,000 IU/L) presenting to our institutions within the time period of the trial. These were women not recruited to the trial, (either not approached or declined participation, see Fig. 1) but instead, were treated medically with methotrexate.

Table 2 shows their baseline characteristics. The median (range) pre-treatment serum hCG concentrations was 2083 (1058–4985) IU/L and were no different to pre-treatment hCG concentrations of trial participants (p = 0.71). Seven cases (22%) had a pre-treatment serum hCG concentrations >3000 IU/L. Thus, baseline hCG levels appeared comparable to the 28 trial participants.

**Table 2:** – Baseline characteristics of a contemporaneous cohort (n = 32).

Characteristic	
Age – (median and IQR)	
Previous live births – n (%)	14 (44%)
Body mass index – (median and IQR)^a^	25 (22–28)
Previous ectopic pregnancy - n (%)	3 (9%)
Self reported past history of pelvic infection, or a positive Chlamydia test – n (%)^b^	6 (18%)
Conception by assisted reproductive technologies – n (%)	2 (6%)
Smoker – n (%)	
Never	24 (75%)
Past smoker	4 (12.5%)
Current smoker	4 (12.5%)

a Body mass index was available for 26 women.

b Women who presented to the Melbourne Hospitals (but not Edinburgh) were offered routine chlamydia testing during their treatment for their ectopic pregnancy, where 3 tested positive.

In this contemporaneous cohort, medical management with either one or two doses of methotrexate resolved the ectopic pregnancy without the need for surgery in 66% of cases (n = 21), lower than 86% rate of success seen among the trial cohort although the comparison was not statistically significant (p = 0.08).

Up until February 2015 (the final date we had permission from ethics to contact women before we closed this study) there had been seven subsequent spontaneous pregnancies in our trial cohort. Five had been successful intrauterine pregnancies, one had a miscarriage and another was diagnosed with another tubal ectopic pregnancy that was treated surgically.

**Fig. 2:**
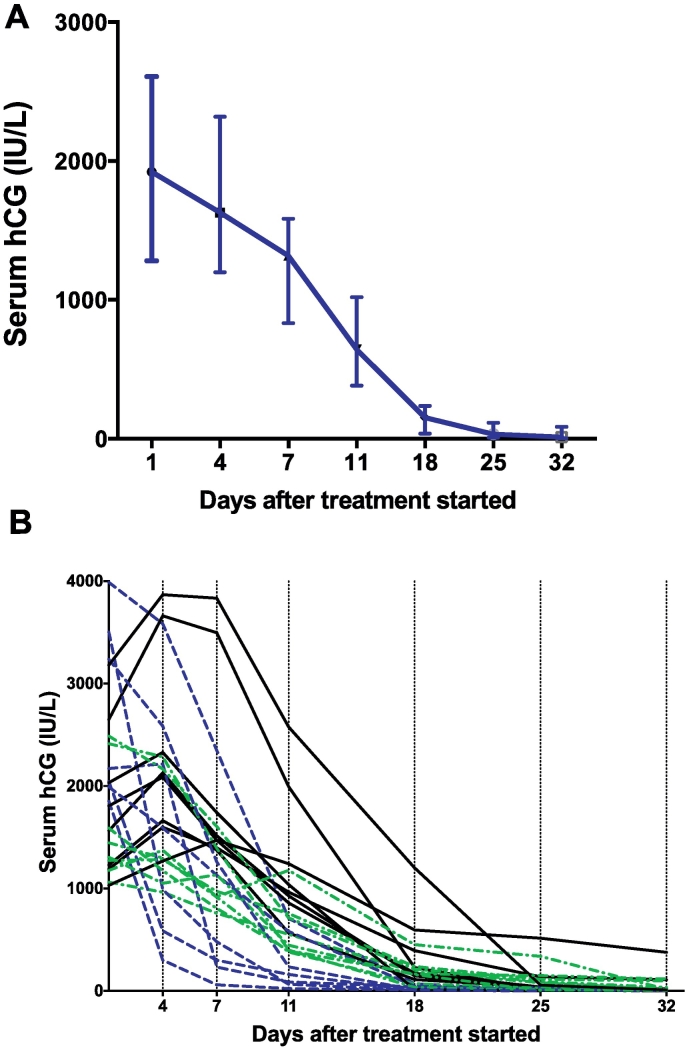
Serum hCG concentrations among the 24 participants who were successfully treated in the trial. Median and interquartile range shown. A) Aggregate serum hCG levels. Median and interquartile range shown. B) Serum hCG of individual participants. Black solid line – participants who had an initial rise in serum hCG before a decline. Green dotted line – participants who had a slow but steady decline in serum hCG levels. Blue dotted line – participants who had an acute fall in serum hCG levels.

The median (±range) time to resolution for the 24 participants successfully treated with combination methotrexate and gefitinib was 32 (18–67) days. Serum hCG levels among these 24 participants are illustrated in Fig. 2A. Eight participants had a transient increase in their serum hCG concentrations before they declined (Fig. 2B – black solid line) and seven experienced a steep decline in serum hCG concentrations from the commencement of treatment (Fig. 2B – dotted blue line). Serum hCG concentrations declined between days 4 and 7 after the start of treatment among 22 out of the 24 (92%) participants who were successfully treated.

## 4 Discussion

Intramuscular methotrexate with seven daily tablets of gefitinib successfully resolved 86% of larger tubal ectopic pregnancies (hCG 1000–10,000 IU/L) without the need for ‘rescue’ surgery. Our pre-specified power calculation suggests this treatment is at least 70% effective. The treatment commonly causes skin rash and diarrhoea but there appeared to be no serious adverse events attributable to the study medication. Furthermore, seven participants subsequently had spontaneous pregnancies.

The treatment trended towards being more effective when the rates of treatment success were compared to a group of 32 women who were administered methotrexate where 66% had treatment success. Of note, the numbers we obtained who were administered methotrexate alone were small. Despite this, there appeared to be a strong trend and it raises the possibility that the combination treatment may be more efficacious (66% vs 86%, p = 0.08) than administering methotrexate alone but we were underpowered to detect this. Whether or not this is in fact truly the case requires a randomised clinical trial but this data supports the case to undertake one.

A strength of our study is that it was designed to examine the efficacy of gefitinib and methotrexate to treat larger ectopic pregnancies. Guidelines published by The Royal College of Obstetricians and Gynaecologists [13] that were current at time we recruited women to our study recommended women with an ectopic pregnancy and a pre-treatment serum hCG < 3000 IU/L could be offered medical management but for those above this threshold, surgery should be considered. Thus, a number of our trial participants would have proceeded to surgery if they were offered regular clinical care following our existing hospital protocols. We note that since we completed this study, new guidelines on the management of ectopic pregnancy published by RCOG in 2016 now suggests the use of methotrexate may be suitable for ectopic pregnancies with a pre-treatment hCG up to 5000 IU/L [14].

Furthermore, we also excluded smaller ectopic pregnancies with a pre-treatment serum hCG < 1000 IU/L. Inclusion of such cases would improve rates of success but dilute our ability to examine whether this treatment is indeed effective in resolving larger ectopic pregnancies.

A limitation to our study is that it was a single arm design. We were keen to test this intervention on larger ectopic pregnancies, including those that would have been treated surgically. Therefore, there was no obvious comparison group as it would have been unethical to mandate medical treatment using methotrexate alone for those with larger ectopic pregnancies where it is known rates of success are perhaps unacceptably low.

We previously published a phase I trial where we treated 12 participants [5] with gefitinib and methotrexate. In contrast to the present study, the prior report recruited women with a pre-treatment serum hCG < 3000 IU/L (where six had a pre-treatment serum hCG < 1000 IU/L and the remaining had a serum hCG between 1000 and 3000 IU/L). 85% (10/12) were successfully treated with the combination treatment. In this present study there was a 86% success rate among the entire cohort.

We also published a case series where we treated eight cases of extra-tubal ectopic pregnancies [6]. Five were in the cornua and two were embedded in an old caesarean section scar. Five had a pre-treatment serum hCG > 5000 IU/L. All eight were successfully treated. Other than these there are no other reports examining the use of gefitinib to treat ectopic pregnancies.

As stated in our published protocol, we did not envisage this would be the final trial to determine whether combination gefitinib and methotrexate should be used clinically to treat ectopic pregnancies [7]. However, this phase II study has achieved the stated primary outcome suggesting the efficacy of this treatment to treat these larger tubal ectopic pregnancies is greater that 70%. In light of the data obtained in this phase II trial, we believe it supports progressing to a phase III randomised trial.

A systematic review and meta-analysis analysing data from two clinical trials [15] concluded that the cost-effectiveness of medical treatment with methotrexate drops significantly with higher (>1500 IU/L) pre-treatment serum hCG and that laparoscopic excision remains the most effective treatment for ectopic pregnancy [16]. Reasons for the increased costs arising from medical management is the expense of outpatient visits and that some participants will still require surgery after a period of observation. If gefitinib and methotrexate where shown to be more effective in a phase III clinical trial than methotrexate alone and resolves them quicker (resulting in less outpatient visits), it may also be worthwhile comparing the costs of the two treatments.

We have now commenced a phase III trial where we plan to randomise 328 women diagnosed with an ectopic pregnancy with an pre-treatment serum hCG <5000 IU/L to either methotrexate alone, or methotrexate and seven daily oral tablets of gefitinib (GEM III trial; EU Clinical Trials register: 2015-005013-76). We selected a pre-treatment serum hCG cut-off of 5000 IU/L, as this is a threshold advocated by the National Institute for Health and Care Excellence (NICE) [17], the American College of Obstetricians and Gynecologists [18] and also the Royal College of Obstetrician and Gynecologists [14] and makes it ethical to perform a placebo controlled trial comparing the combination with methotrexate treatment alone. Our primary outcome for this new trial is the need for surgical intervention in the resolution of the tubal ectopic pregnancy. Should our phase III randomised trial yield a positive result, this may represent sufficient evidence to show adding gefitinib to the current methotrexate protocol improves the efficacy of medical management, and this could become standard of care.

In conclusion, combination gefitinib and methotrexate was 86% (and at least 70% effective) in resolving ectopic pregnancies with a pre-treatment serum hCG 1000–10,000 IU/L. Transient diarrhoea and rash are common side effects. This may be a new medical treatment to treat most stable ectopic pregnancies, but a randomised clinical trial needs to be completed before this can be used in the clinic.

## Funding Sources

This work was supported by an NHMRC Grant (#1008276) to ST, TJ and EW, and an MRC Centenary Award (G0802808) to AH. The funders had no role study design, collection, analysis, and interpretation of data; in the writing of the report or in the decision to submit the paper.

## Declaration of Interests

TJ, and ST are joint holders of patents that relate to the use of EGFR inhibition in treating ectopic pregnancies. There are no other conflicts of interest to disclose.

## Author Contributions

MMS set up the trial, recruited participants in Melbourne, analysed the data and drafted the paper. S.T. helped design the trial, obtained funding, supervised recruitment in Melbourne, provided oversight for the trial, helped analyse the data and drafted the initial versions of the paper. A.D. helped design the trial and co-ordinated the trial in Edinburgh, supervising recruitment. J.M. helped design the trial, providing statistical input in both the design and in the final analysis. TGJ provided input in the analysis and drafting of the paper. PN, MR, and NK helped with recruitment in Melbourne. EW obtained funding for the trial. SB provided input in the design and drafting of the paper. WCD helped with the design of the trial, provided supervision of the trial in Edinburgh. AWH designed the protocol, helped set up the trial in Edinburgh, supervised recruitment in Edinburgh and provided oversight for the trial with ST, helped analyse the data and drafted the initial versions of the paper. All authors contributed to the drafting of the paper.

## References

[1] Anon (1995). Ectopic pregnancy: United States, 1990–1992.

[2] The Confidential Enquiry into Maternal and Child Health (CEMACH) (2007). Saving mother's lives: Reviewing maternal deaths to make motherhood safer 2003–2005. The seventh report on confidential enquiries into maternal deaths in the United Kingdom London.

[3] Jurkovic D., Wilkinson H. (2011). Diagnosis and management of ectopic pregnancy. BMJ.

[4] Nilsson U.W., Johns T.G., Wilmann T. (2013). Effects of gefitinib, an epidermal growth factor receptor inhibitor, on human placental cell growth. Obstet Gynecol.

[5] Skubisz M.M., Horne A.W., Johns T.G. (2013). Combination gefitinib and methotrexate compared with methotrexate alone to treat ectopic pregnancy. Obstet Gynecol.

[6] Horne A.W., Skubisz M.M., Tong S. (2014). Combination gefitinib and methotrexate treatment for non-tubal ectopic pregnancies: A case series. Hum Reprod.

[7] Horne A.W., Skubisz M.M., Doust A. (2013). Phase II single arm open label multicentre clinical trial to evaluate the efficacy and side effects of a combination of gefitinib and methotrexate to treat tubal ectopic pregnancies (GEM II): Study protocol. BMJ Open.

[8] A'Hern R.P. (2001). Sample size tables for exact single-stage phase II designs. Stat Med.

[9] Skubisz M., Dutton P., Duncan W.C., Horne A.W., Tong S. (2013). Using a decline in serum hCG between days 0–4 to predict ectopic pregnancy treatment success after single-dose methotrexate: A retrospective cohort study. BMC Pregnancy Childbirth.

[10] Avcioglu S.N., Altinkaya S.O., Kucuk M., Demircan Sezer S., Yuksel H. (2014). Predictors of success of different treatment modalities for management of ectopic pregnancy. Obstet Gynecol Int.

[11] Cataldo V.D., Gibbons D.L., Perez-Soler R., Quintas-Cardama A. (2011). Treatment of non-small-cell lung cancer with erlotinib or gefitinib. N Engl J Med.

[12] Stovall T.G., Ling F.W., Gray L.A. (1991). Single-dose methotrexate for treatment of ectopic pregnancy. Obstet Gynecol.

[13] Royal College of Obstetricians and Gynaecologists (2004). The management of tubal pregnancy. GreenTop guideline No 21 (Updated 2010).

[14] Royal College of Obstetricians and Gynaecologists (2016). Diagnosis and management of ectopic pregnancy. Green-top guideline No.21.

[15] Sowter M.C., Farquhar C.M., Gudex G. (2001). An economic evaluation of single dose systemic methotrexate and laparoscopic surgery for the treatment of unruptured ectopic pregnancy. BJOG.

[16] Mol F., Mol B.W., Ankum W.M., van der Veen F., Hajenius P.J. (2008). Current evidence on surgery, systemic methotrexate and expectant management in the treatment of tubal ectopic pregnancy: A systematic review and meta-analysis. Hum Reprod Update.

[17] Ectopic pregnancy and miscarriage (2012). Diagnosis and initial management in early pregnancy of ectopic pregnancy and miscarriage. NICE clinical guidelines.

[18] American College of O, Gynecologists (2008). Medical management of ectopic pregnancy. Obstet Gynecol.

